# Tussis for Obstructive Stridor, an Impromptu Cure: A Rare Case of Spontaneous Resolution of Obstructive Fibrinous Tracheal Pseudomembrane (OFTP)

**DOI:** 10.7759/cureus.12699

**Published:** 2021-01-14

**Authors:** Rama Kanth Pata, Oday Alhafidh, Abolfazl Ahmady, Roudabeh Kiani

**Affiliations:** 1 Internal Medicine, Interfaith Medical Center, Brooklyn, USA; 2 Pulmonary Medicine, New York University (NYU) Langone Hospital, Brooklyn, USA; 3 Pulmonary Medicine, Interfaith Medical Center, Brooklyn, USA

**Keywords:** intubation, obstructive fibrinous tracheal pseudomembrane, oftp, stridor, tussis

## Abstract

Obstructive fibrinous tracheal pseudomembrane (OFTP) is a relatively rare complication of endotracheal intubation. OFTP, which may cause fatal airway obstruction, is perhaps more common than reported in the literature. Although little is known about the mechanisms that play a role in the development of OFTP, it is hypothesized that OFTP results from ischemic injury to tracheal mucosa. Diagnosis is made using rigid bronchoscopy, which is also used for excision and removal of the pseudomembrane. Here we present a case of OFTP in a patient who was intubated for three days, developed symptoms three days after extubation, and ultimately spontaneously expelled the tracheal pseudomembrane.

## Introduction

Obstructive fibrinous tracheal pseudomembrane (OFTP) is a relatively rare complication of endotracheal intubation. Although little is known about the mechanisms that play a role in the development of OFTP, it is hypothesized that flawed intubation or extubation causes ischemic injury to the tracheal mucosa. This ischemia may be a result of an overinflated cuff of the endotracheal tube (ETT) or hypoperfusion and accentuated by a caustic injury caused by vomiting [1]. This injury induces the production of growth factors and initiates a cascade leading to abnormal regeneration [2]. Subsequently, a fibrous membrane forms that partially or completely obstructs the tracheal lumen [2,3]. Consequently, patients develop dyspnea and stridor several days after extubation [1,3,4]. Rigid bronchoscopy is usually used for visualization and excision of the pseudomembrane [1,3,4]

## Case presentation

A 31-year-old gentleman with a recent history of a motor vehicle accident was brought in by the emergency medical services accompanied by his mother due to breathing difficulty. He reported that five days ago, he was involved in a motor vehicle accident, in which he sustained head trauma and underwent invasive mechanical ventilation. He was extubated two days later and stayed under observation for three days. Subsequently, he signed out against medical advice from the hospital; however, on the way back home, he developed "difficulty breathing with a sensation of something being stuck in his throat". His mother contacted the emergency medical services, and he came to the emergency department.

His medical history was otherwise not significant, except for the history of smoking and occasional alcohol consumption. His physical examination was benign, vitals were stable (Table 1), and blood tests were normal (Table 2). A chest X-ray showed no abnormalities, and a head CT scan without contrast showed no evidence of acute intracranial hemorrhage, infarct, or extra-axial fluid collection.

**Table 1:** Patient’s vital signs on presentation F: Fahrenheit; bpm: beats per minute; mmHg: millimeter of mercury

Vital sign parameter	Result
Temperature (F)	99.5
pulse (bpm)	100
blood pressure (mmHg)	135/65
respiratory rate (per min)	16
peripheral oxygen saturation	100% on room air

**Table 2:** Blood test results on presentation WBC: white blood cell count; Hgb: hemoglobin; Hct: hematocrit; PT: prothrombin time; INR: international normalized ratio; APTT: activated partial thromboplastin time; Na: Sodium; K: Potassium; Cl: Chloride; BUN: blood urea nitrogen; Cr: creatinine.

Lab test	Result
WBC ( /uL)	8800
Hgb (g/dL)	11.9
Hct (%)	35.4
Platelet (/uL)	372,000
PT (Seconds)	12.3
INR	1.07
APTT (Seconds)	32.3
D-dimer (ng/ml)	3648
Na (mmol/L)	141
K (mmol/L)	4.0
Cl (mmol/L)	104
Carbon Dioxide (mEq/L)	25
Anion gap	12
BUN (mg/dL)	8.8
Cr (mg/dL)	0.74
Troponin I (ng/mL)	0.00

**Figure 1:**
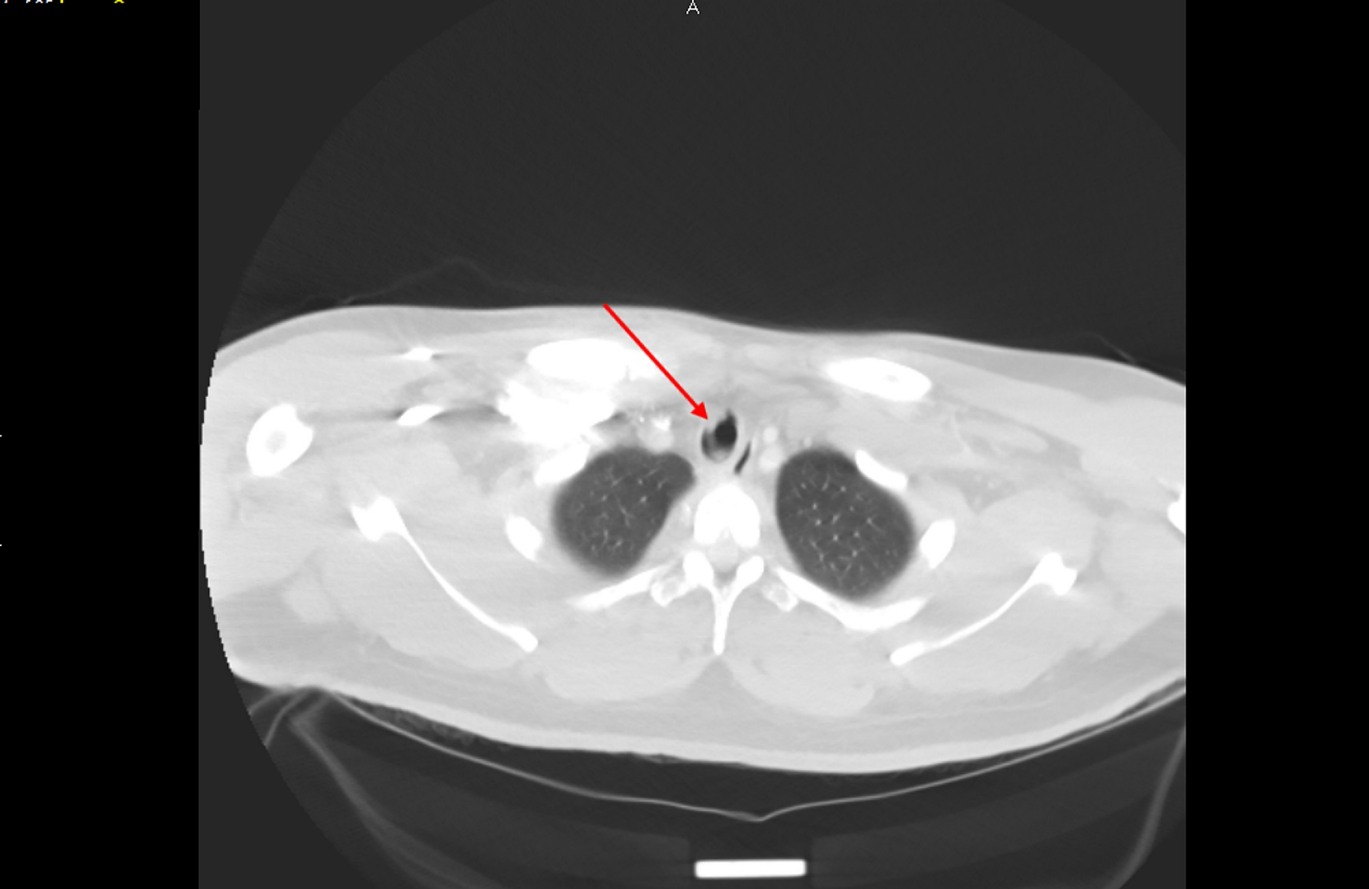
Chest CT scan suggesting the presence of a membranous structure in the trachea (arrow)

**Figure 2:**
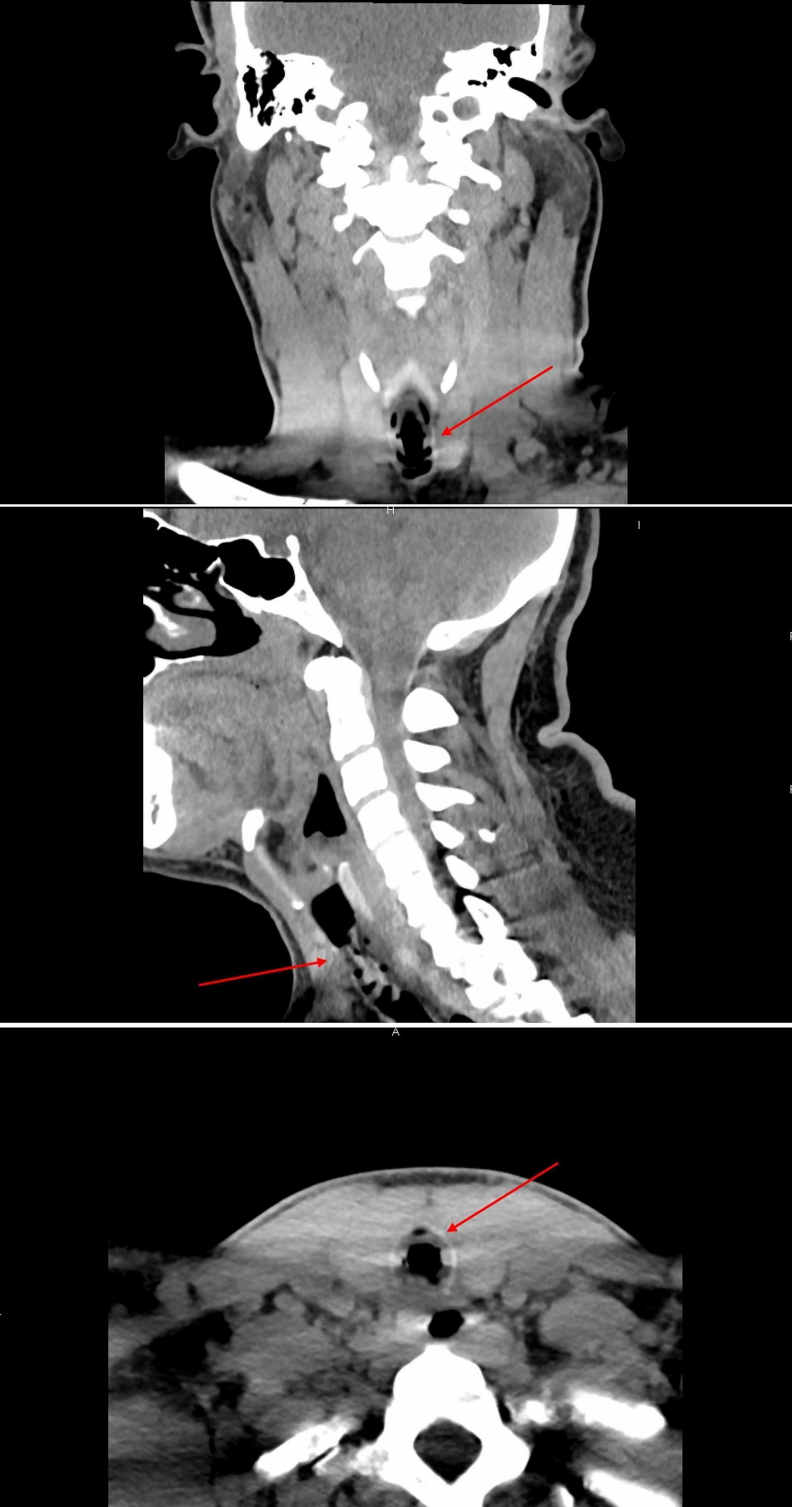
Soft Tissue Neck CT scan showing prominent airway narrowing throughout the nasopharynx and oropharynx based on the prominence of the lymphoid soft tissues and markedly abnormal trachea below the cricoid cartilage with the presence of stenosis (arrows)

**Figure 3:**
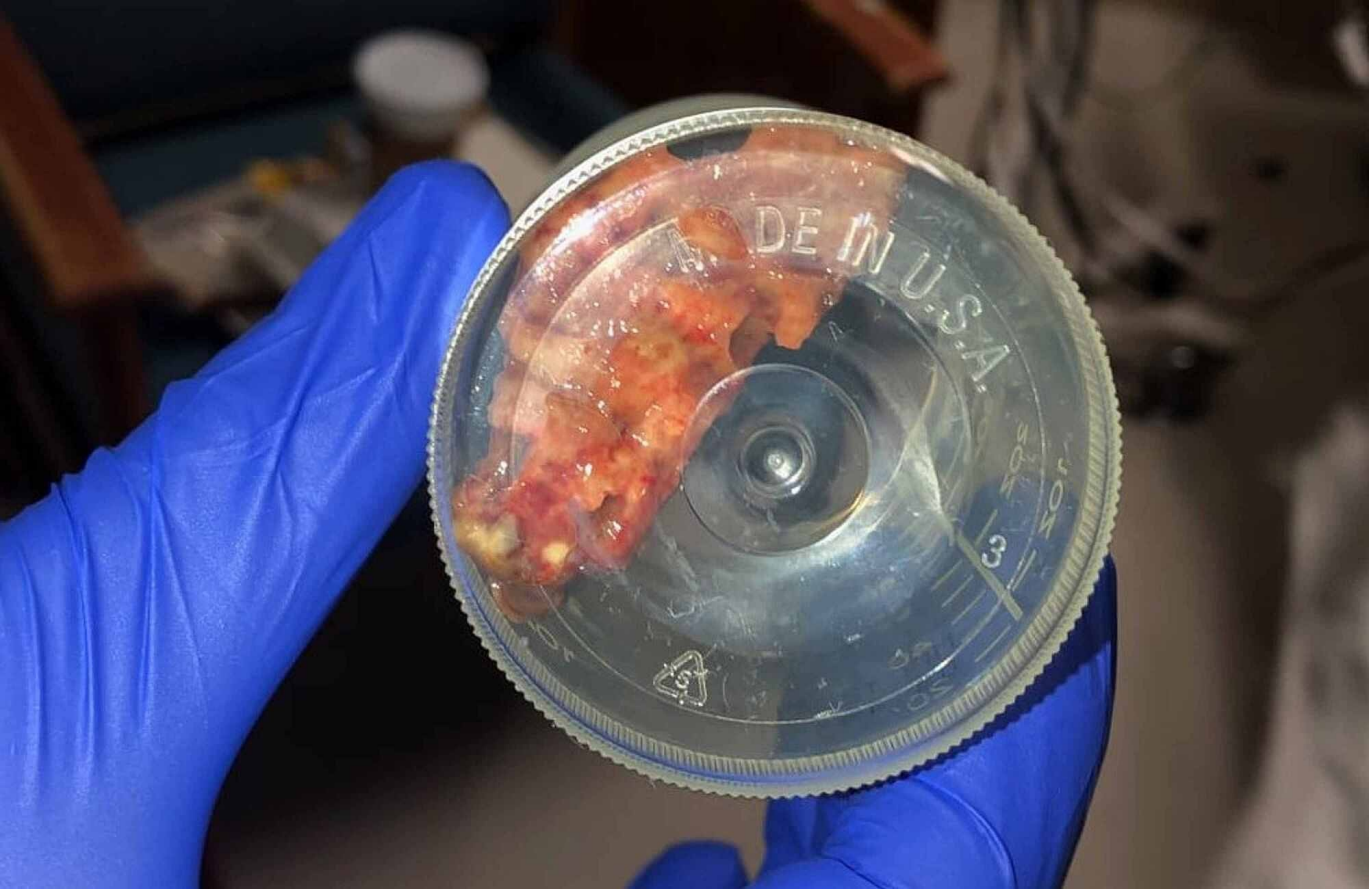
Specimen coughed out by the patient A single irregular fragment of friable material measuring 3 cm in length and 1 cm wide and 0.5 cm in thickness was coughed out by the patient.

**Figure 4:**
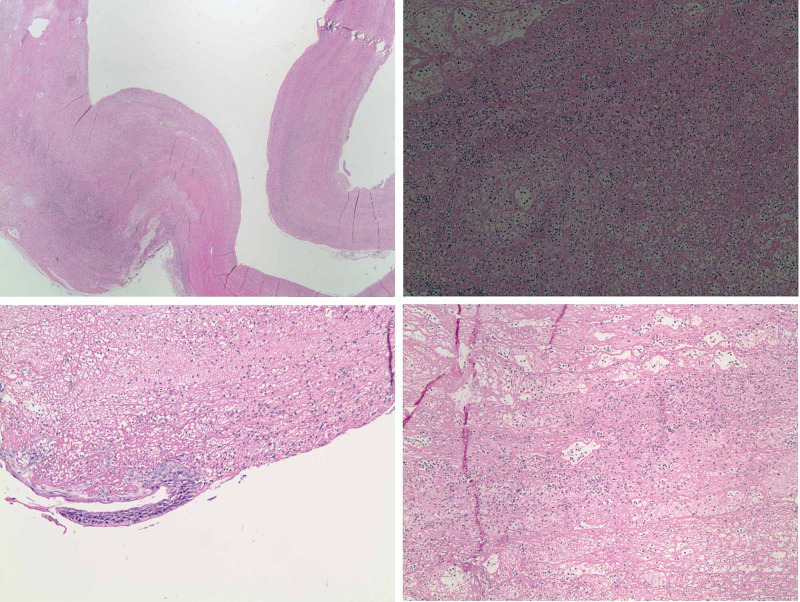
Histopathology of the specimen coughed out by patient showing a fragment of fibrin mixed with acute inflammatory cells and few degenerated epithelial cells

**Figure 5:**
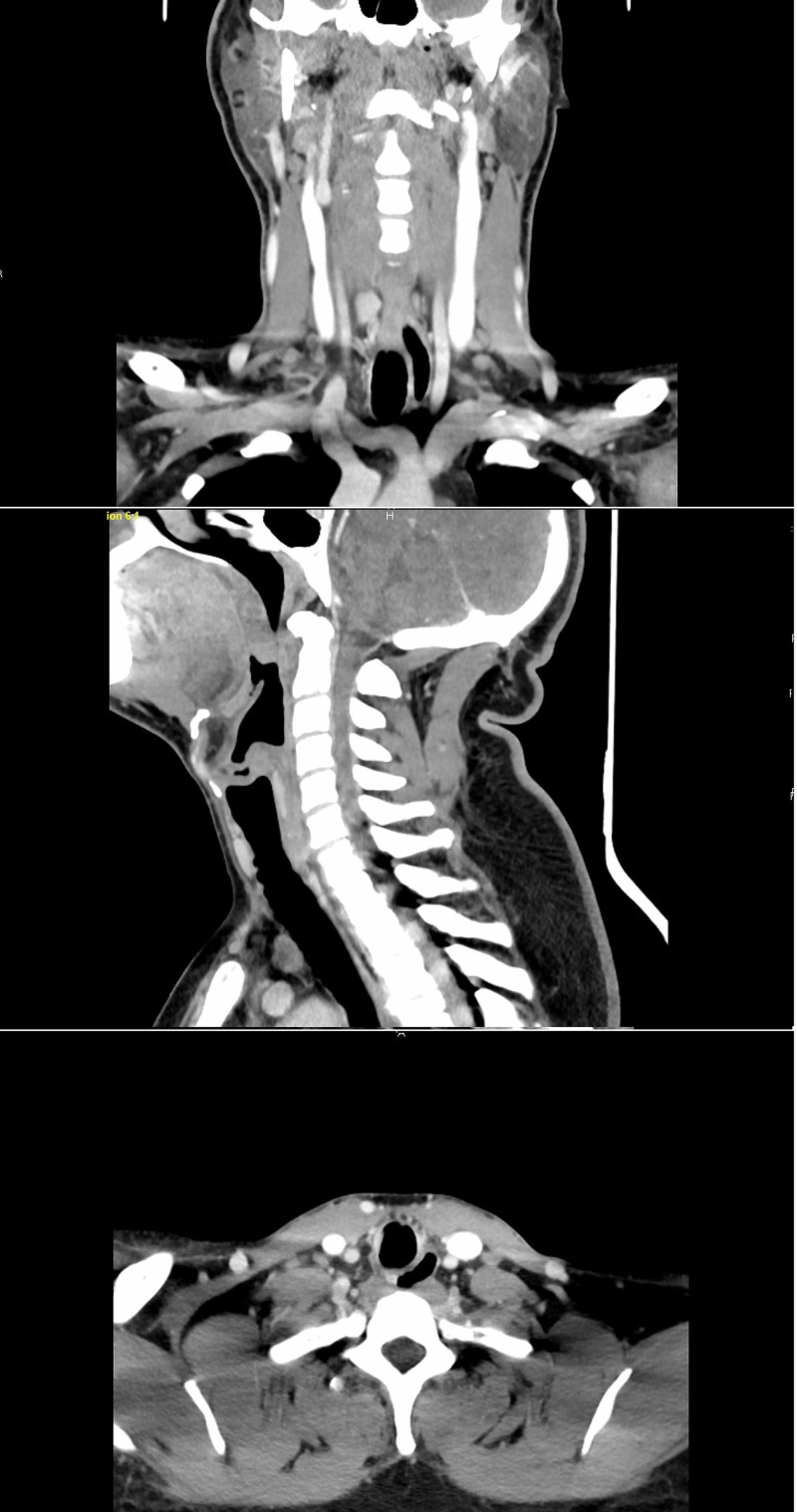
Follow up soft tissue neck CT scan confirming complete expulsion of the obstructive lesion

Shortly after arrival, he developed stridor, shortness of breath, tachycardia, and tachypnea (Table 3), at which point, he was started on a course of racemic epinephrine, albuterol, and dexamethasone, with mild improvement in symptoms. As improvements were not satisfactory, a chest CT scan with contrast was performed, which showed no evidence of acute pulmonary embolism. However, a membranous structure in the trachea was incidentally discovered (Figure 1). Subsequently, a CT scan of the neck soft tissue revealed prominent airway narrowing throughout the nasopharynx and oropharynx based on the prominence of the lymphoid soft tissues and markedly abnormal trachea below the cricoid cartilage with luminal narrowing (Figure 2). Consequently, he was admitted to the intensive care unit, and in the meantime, emergency bronchoscopy was planned for direct visualization and possible extraction of the tracheal pseudomembrane. However, a short time later he coughed up a single irregular fragment of friable material measuring 3 cm in length, 1 cm width, and 0.5 cm in thickness (Figure 3). Histopathology of the specimen showed a fragment of fibrin mixed with acute inflammatory cells and few degenerated epithelial cells (Figure 4), and a follow-up CT scan of the neck soft tissue confirmed complete expulsion of the obstructive lesion (Figure 5).

**Table 3:** Patient’s vitals and arterial blood gas results at the onset of respiratory distress in the emergency room pCO2: partial pressure of carbon dioxide; pO2: partial pressure of oxygen; FiO2: the fraction of inspired oxygen

Vital sign/arterial blood gas parameter	Result
Pulse (bpm)	119
Blood pressure (mmHg)	130/75
Respiratory rate (per min)	30
PH	7.410
pCO2	41.4
pO2	181
Oxygen saturation	99.2
O2 delivery method	Room air
FiO2	21%

## Discussion

The first case of post-intubation tracheal pseudomembrane formation, also known as Obstructive Fibrinous Tracheal Pseudomembrane (OFTP), was reported in the literature by Sigrist et al. in 1981 [5], but the term OFTP was proposed by Deslée et al. in 2000 [6]. It is well established that tracheal pseudomembrane formation is associated with multiple fungi, bacteria, and viruses, most notably diphtheria [3,4]. However, OFTP is rarely diagnosed and reported; hence, the incidence of OFTP is unknown [4]. Perhaps the reason for this underdiagnosis is that, due to the acute onset of symptoms and respiratory failure, most patients are intubated before the diagnosis of OFTP via bronchoscopic visualization [1].

The exact etiology of OFTP is not clearly known. One hypothesis postulates that the pathophysiology behind this disease is secondary to high pressure in an endotracheal tube cuff and resultant tracheal mucosal ischemia [1,3]. Casoni and colleagues [7] reported a case of OFTP development in a patient who was intubated by a high-pressure, low-volume cuff for 18 hours. Kang and colleagues [8] presented a case of OFTP development where endotracheal tube cuff pressure was maintained at 15 cm H2O during intubation; however, traumatic intubation aggravated by caustic injury due to vomiting was described as the etiologic factor of OFTP development. Furthermore, Rice and colleagues presented two patients with hypoperfusion-induced development of OFTP, suggesting that hypotension and hypoperfusion may cause ischemic insult to the tracheal mucosa even with a high-volume, low-pressure cuff [9]. Increased pressure, trauma, or hypoperfusion cause ischemia and inflammation of the tracheal mucosa and submucosa [1,4]. These insults induce the generation of growth factors, commencing a cascade that promotes abnormal regeneration [2]. Ultimately, hemorrhagic infarction of the submucosa, with profuse infiltration by polymorphonuclear cells and fibrinous exudate [1,4], leads to the formation of a fibrous membrane forms that partially or completely obstruct the tracheal lumen [2,3].

Although most patients present with dyspnea and stridor after extubation [1,3,4], similar to our case, other presentations are also possible, such as paroxysmal stridor, as described by Patolia and colleagues [1] in a patient with a valve-like obstruction of the trachea and severe respiratory failure without stridor, as airflow is poor in the trachea [1]. The onset of symptoms has been reported to be from a few hours up to nine days after extubation [1]. In our case, the patient’s symptoms started three days after extubation.

There are several more frequent causes of dyspnea and stridor after extubation, such as bronchial asthma, vocal cord dysfunction, glottic edema, laryngospasm, or laryngeal edema [3,4]; however, because OFTP is potentially fatal, it is crucial to suspect this condition in such patients. Diagnosis is made by bronchoscopy to examine the area to rule out laryngeal edema or paralysis and visualize the obstructive membrane [1,3,4]. However, in our case, suspicion was raised by the incidental neck findings seen on the chest CT scan, and the diagnosis was made by visualization of the membrane using a neck soft tissue CT scan.

Removal of the obstructive lesion using rigid bronchoscopy is recommended [1,3,4], and dilatation and laser/stent placement are not required [1]. However, it should be noted that in some cases, the lesion spontaneously expels, as in our case. In these cases, even though the condition does not recur [1], it is important to perform a follow-up neck soft tissue CT scan or bronchoscopy to ensure the expulsion of the whole membrane.

## Conclusions

Because OFTP is potentially fatal, and to prevent unnecessary tracheostomy, it is crucial to suspect this condition in patients who develop stridor and dyspnea shortly after endotracheal extubation. Fiberoptic bronchoscopy, preferably rigid bronchoscopy, is recommended for diagnosis and removal of the obstructive lesion.
